# Hospital length of stay variation and comorbidity of mental illness: a retrospective study of five common chronic medical conditions

**DOI:** 10.1186/s12913-018-3316-2

**Published:** 2018-06-27

**Authors:** Nazlee Siddiqui, Mitchell Dwyer, Jim Stankovich, Gregory Peterson, David Greenfield, Lei Si, Leigh Kinsman

**Affiliations:** 1 Australian Institute of Health Services Management (AIHSM), Tasmanian School of Business and Economics, University of Tasmania, Rozelle campus, Cnr Glover and Church Streets, Sydney, NSW 2039 Australia; 2 School of Health Sciences, University of Tasmania, Tasmania, Australia; 3 Health Services Innovation Tasmania, School of Medicine, University of Tasmania, Tasmania, Australia; 4 Centre for the Health Economy, Macquarie University, Sydney, Australia; 5 Conjoint appointment, University of Tasmania and Tasmanian Health Service (North), Tasmania, Australia

**Keywords:** Comorbidities, LOS variation, hospital

## Abstract

**Background:**

With the increasing burden of mental illness globally, it is becoming common for hospitalised patients with chronic medical conditions to have a comorbidity of mental illness. This combination could prolong length of stay (LOS) of this patient cohort. We conducted an investigation in Tasmania, Australian hospitals to characterise this cohort and assess if co-morbidity of mental illness is a distinguishing factor that generates LOS variation across different chronic medical conditions.

**Methods:**

The retrospective study analysed 16,898 admissions of patients with a primary diagnosis of one of five chronic medical conditions: lung or colorectal cancer, chronic obstructive pulmonary disease (COPD), type II diabetes, ischaemic heart disease (IHD) and stroke. Data were from July 2010 to June 2015, across four hospitals that collectively cover 95% of public hospital admissions in Tasmania, Australia. Descriptive statistics were used to compare characteristics of patients between the scenarios of with and without co-morbidity of mental illness. We used negative binomial regression models to assess whether co-morbidity of mental illness, along with its sub-types, after adjustment for potential confounding variables, associated with LOS variation in patients of each medical condition. Based on the adjusted LOS variation, we estimated differences in bed days’ use between patients with and without comorbidity of mental illness.

**Results:**

Patients with co-morbidity of mental illness were significantly younger in comparison to patients without mental illness. With each medical condition, patients with comorbidity of mental illness had incurred higher bed days’ use than for those without mental illness. In cancer and stroke cohorts, co-morbidity of mental illness unfavourably affected the LOS variation by as high as 97% (CI: 49.9%–159%) and 109% (78%–146%), respectively. Though mental and behavioural disorders due to psychoactive substances was a dominant sub-type of mental illness across the medical conditions, it contributed significant unfavourable LOS variation only in the stroke patients i.e. 36.3% (CI: 16.2%–59.9%).

**Conclusions:**

Mental illness consistently produced unfavourable LOS variation. Upskilling of healthcare teams and greater reporting and analysis of LOS variation for this patient cohort, and the sub-cohorts within it, are necessary to provide improved medical care and achieve system efficiencies.

## Background

It has been reported that hospitalised patients with chronic medical conditions and a comorbidity of mental illness can experience a longer length of stay (LOS), including patients with chronic obstructive pulmonary disease (COPD) [1], diabetes [2, 3], ischaemic heart disease (IHD) [4], and stroke [5]. This outcome is poor for both patients and the health system that strives to care for them. Longer LOS reflects a compromise to this vulnerable population’s quality of life and care experience, and highlights health system inefficiencies [6, 7]. This is a growing problem internationally, with ongoing increases in hospitalised patients having a comorbidity of mental illness [8–11].

A key concern is that many health jurisdictions do not have a detailed knowledge about characteristics of patients with chronic medical disease and a comorbidity of mental illness [12]. The knowledge deficit extends to a lack of understanding about LOS variation and improvement strategies for this cohort. LOS variation is a parameter to show differences in average LOS within the same condition between peer hospitals [13–15]. LOS variation is a well-accepted parameter to assess efficiency in hospitals in Australia [13], the United States [16] and other Organisation for Economic Cooperation and Developed (OECD) countries [17, 18]. As unfavourable LOS variation can indicate excess bed days’ use that could have catered for more admitted patients [7]. However, it is not standard practice to report LOS variation of patients with chronic medical conditions and a comorbidity of mental illness separate from that of hospitalised patients with a chronic medical condition only.

Furthermore, existing published information about LOS for patients with chronic medical conditions and a comorbidity of mental illness is limited. Studies of LOS variation often have only examined one or two medical conditions [4, 19, 20] and did not control for patient characteristics or were limited to a single hospital setting [5]. To improve care outcomes and promote efficient functioning of the health system, we need the capability to more specifically identify and manage hospitalised patients with chronic medical conditions and a comorbidity of mental illness [11, 21–23].

To address this issue, we conducted a study investigating two research questions: first, are there differences in characteristics of patients with chronic medical condition with and without a comorbidity of mental illness? Second, is a comorbidity of mental illness a distinguishing factor that generates LOS variation across different chronic medical conditions? Cancer (lung and colorectal only), COPD, diabetes (type 2), IHD and stroke are five chronic conditions that reflect about 20% of the burden of disease in Tasmania, Australia [24]. These five conditions are also in the list of top 10 leading causes of death in the world [25]. Accordingly, we investigated the two research questions with patients having these five chronic medical conditions in the context of Tasmanian public hospitals.

## Methods

### Data and sources

The data related to patients admitted to any of the four major public hospitals in Tasmania, Australia (i.e. Royal Hobart Hospital, Launceston General Hospital, Mersey Community Hospital, and North West Regional Hospital). These four hospitals are collectively responsible for 95% of all public hospital admissions in the Tasmanian state that has 500,000 inhabitants [26, 27]. In this retrospective study, we extracted de-identified demographic and clinical information for hospital admissions that occurred between 1 July 2010 and 30 June 2015. We accessed the “Admitted patient care National Minimum Data Set (NMDS)” for these admissions as provided by the Tasmanian Department of Health and Human Services. The admitted patient care NMDS is a minimum set of data elements for inpatient care that health authorities around Australia mandatorily report to support national collation [28, 29]. We used specific fields from this dataset for each admission: hospital; patient sex, date of birth, date of admission and discharge (or separation), area of residence; and a sequence of international classification of disease (ICD)-10 codes extracted from medical records by clinical coders. One of these ICD-10 codes from each admission was designated as the primary diagnosis.

### Study measures

We analysed data for admissions where the primary diagnosis was one of five chronic medical conditions: cancer (lung and colorectal only), COPD, diabetes (type 2), IHD or stroke. The next step involved further screening of the patients, to identify those with a mental illness ICD-10 code entered as a secondary diagnosis during the course of the admission. Table 1 shows the ICD-10 codes used to define each of the chronic medical conditions and mental illnesses.

**Table 1:** Coverage of chronic medical conditions and mental illness in this study

Illnesses	ICD-10 codes, in accordance to International statistical classification of diseases (ICD)
Chronic medical conditions as primary diagnosis	Cancer: Lung cancer “C33”,“C34”; Colorectal Cancer “C18”,“C19”,“C20”,“C21”
	COPD: “J44.”
	Type II Diabetes: “E11”
	IHD: “I20”,“I21”,“I22”,“I23”, “I24”, “I25”
	Stroke: “I60”,“I61”,“I62”,“I63”,“I64”,“I65”,“I66”,“I67”,“I68”,“I69”,“G45”,“G46”
Mental illnesses as secondary diagnosis	“F04”, “F06”, “F07”, “F09”, “F10–F19”, “F20–F29”, “F30–F39”, “F40–F48”, “F50–F59”, “F60–F69”, “F70–F79”, “F80–F89”, “F90–F99”

Details of medical conditions and mental illnesses for each ICD-10 code are accessible at World Health Organisation (WHO) website [30].

Consistent with previous reporting of mental illness, we omitted patients with dementia (F00-F03) [31] and delirium (F05) [31, 32] from the mental illness cohort. Complete information on all variables was available for over 99% of admissions, making it appropriate to exclude a case (i.e. patient admission) from analysis only if the required data for the specific analysis was missing.

Other variables computed from the extracted data were LOS, bed days’ use, age, socioeconomic status (SES), and presence of an additional comorbidity. The measurement of the primary outcome measure, LOS, was in whole days, equal to date of discharge or separation minus admission date. We coded LOS as zero (0) for admissions where discharge date was the same as admission date. Calculation of bed day’s use, which is another outcome variable, was based on the average percentage increase (p) in the adjusted LOS of the negative binomial regression run in this study. Explanation of this calculation is provided in the statistical analysis section of the paper. Average measures of SES for applicable geographical regions were downloaded from the website of the Australian Bureau of Statistics [33]. This average measure of SES is drawn from the Index of Relative Socio-Economic Advantage and Disadvantage (IRSAD), a weighted average score of an area’s characteristics such as material and social resources [34]. Data for this index is recorded in the Australian census, standardised to a distribution where the mean equals 1000 and standard deviation is 100 [34]. The IRSAD score reflects an area’s average socio-economic status rather than that of individuals living in that area [35]. Higher scores in the index infer higher level of advantage, with areas of higher-than-average socio-economic advantage having scores above 1000.

Charlson comorbidity is a method to identify comorbidities and variation of complexity in patients with a list of 17 comorbidities [36–38]. We used lists of ICD-10 codes validated by Quan et al. [38] to assess the presence or absence of additional Charlson comorbidities in patients. Patients were dichotomised into group of higher complexity when additional Charlson comorbidities were present and vice versa for the group of low complexity. For four of the five chronic medical conditions listed in Table 1 (Type II diabetes, cancer, stroke and COPD), all patients with the condition had at least one of the 17 Charlson comorbidities. Accordingly, we classified patients with these four conditions for presence or absence of additional Charlson comorbidities other than the Charlson comorbidity associated with the primary condition. For example, patients with a primary diagnosis of Type II diabetes were classified into those with and without a Charlson comorbidity other than “diabetes without chronic complication”. Similarly, patients with a primary diagnosis of (a) cancer, (b) stroke and (c) COPD were classified into groups of those with and without a Charlson comorbidity other than (a) “malignancies including lymphoma and leukaemia except malignant neoplasm of the skin”, (b) “cerebrovascular disease” and (c) “chronic pulmonary disease”, respectively. For the fifth condition in Table 1 (IHD), even though there are several cardiovascular comorbidities among the 17 Charlson comorbidities by definition, not all patients with a primary diagnosis of IHD had one of these comorbidities. Hence, patients with a primary diagnosis of IHD were divided simply into those with at least one Charlson comorbidity and those without any Charlson comorbidities.

### Statistical analysis

To address the first research question, we utilised descriptive statistics to compare patients within each chronic medical condition, for scenarios of with and without the comorbidity of mental illness. T-tests were used to compare the means of continuous variables, and Fisher exact tests were used to compare distributions of binary variables.

We addressed the second research question with stages of analyses. Firstly, negative binomial regression was used to test for an independent association between LOS and comorbidity of mental illness. This analysis was run using the glm.nb() command from the “MASS” package in R, which contains functions and datasets associated with Venables and Ripley's Modern Applied Statistics With S [39]. The analysis was adjusted for six potential confounding factors: hospital, financial year of separation or discharge (categorical), presence of Charlson comorbidity, age (5-year categories), gender and SES (treated as a continuous variable). To test whether different subtypes of mental illness were associated with different changes in LOS, the mental illness diagnoses in Table 1 were divided into their blocks as per ICD-10 WHO version [40]: F04–F09 (excluding F05), F10–F19, F20–29, F30–39, F40–F48, F50–F59, F60–F69, F70–F79, F80–F89, F90–98 and F90–F99. In the model for each chronic condition, if codes from a particular block occurred in 10 or more admissions, that block was coded as an additional binary variable (in addition to the binary variable coding of overall presence/absence of mental illness) and added to the regression model. Backwards stepwise regression [35] was then used to remove non-significant blocks until all blocks remaining in the model (if any) had Wald p-values less than 0.05. Changes in the adjusted LOS were calculated for each remaining significant block. The same calculation was applied for all other sub-type of mental illnesses, that is, the group of non-significant blocks and blocks with less than 10 admissions.

The next stage was assessing the independent association of comorbidity of mental illness with LOS, for differences in usage of bed days. This aspect of the analysis required several steps. First, we estimated the average percentage increase (*p*) in adjusted LOS, associated with mental illness (Column 5 in Table 4). This percentage increase *p* is equal to (exp(*b*) – 1) × 100%, where *b* is the coefficient of the mental illness term in the negative binomial regression model. Then, we calculated the difference in bed days’ use, reducing the p to zero, with the assumption of same average LOS for patients of chronic medical conditions, between the scenarios of with and without a comorbidity of mental illness. For example, if we consider n patients having a comorbidity of mental illness with a particular chronic disease (Column 1 in Table 4) and that average LOS for these n patients is t days (Scenario 2 in Column 2 in Table 4). Then, the estimated difference in bed days’ use in five years came to be n*t*p / (1+ p) (Column 6 in Table 4). Similarly, the estimated bed days’ use per patient came to be t*p / (1 + p) (Column 6 in Table 4). The estimated bed days’ use represents the adjusted value, controlling for the influence of the confounding variables.

## Results

The study sample comprised 16,898 admissions across the five chronic disease categories. As shown in Table 2, the most common reason for admission was IHD (*n* = 8,005) and the least common was type 2 diabetes (*n* = 1,299). There was great variation in the proportion of admissions with a mental illness comorbidity between the different chronic medical conditions. For example, the rate of COPD admissions with a comorbidity of mental illness was 23%, while for IHD admissions the rate was only 3%.

**Table 2.** Comparison of patients with chronic medical conditions of scenario 1: admission with no diagnosed secondary mental illness (MI) versus scenario 2: admission with a diagnosed secondary MI

Primary diagnosis recorded during admission	Scenario 1: Without MI	Scenario 2: With MI	Difference between the scenarios
Cancer (Lung and Colorectal) (*n* = 1147)			
Number of admissions (%)	1047 (91.3%)	100 (8.7%)	
Mean age, years (SD)	70.1 (11.7)	67.1 (10.9)	*P* = 0.01^a*^
No. of females (%)	424 (40%)	45 (45%)	*P* = 0.40^b^
Mean Socio-Economic Index for Area^c^ (SD)	929 (68)	925 (67)	*P* = 0.54^a^
No. of admissions with at least one additional CC^d^ (%)	759 (72%)	83 (83%)	*P* = 0.02^b*^
COPD (*n* = 1404)			
Number of admissions (%)	1084 (77%)	320 (23%)	
Mean age, years (SD)	71.6 (10.3)	68.0 (10.8)	*P* < 0.01^a**^
No. of females (%)	547 (50%)	163 (51%)	*P* = 0.90^b^
Mean Socio-Economic Index for Area^c^ (SD)	914 (72)	912 (67)	*P* = 0.57^a^
No. of admissions with at least one additional CC^e^ (%)	305 (28%)	86 (27%)	*P* = 0.67^b^
Diabetes (type 2) (*n* = 1299)			
Number of admissions (%)	1209 (93.1%)	90 (6.9%)	
Mean age, years (SD)	64.5 (14.9)	59.5 (13.7)	*P* < 0.01^a**^
No. of females (%)	390 (32%)	30 (33%)	*P* =0.82^b^
Mean Socio-Economic Index for Area^c^ (SD)	928 (74)	925 (66)	*P* = 0.66^a^
No. of admissions with at least one additional CC^f^ (%)	910 (75%)	74 (82%)	*P* = 0.13^b^
Ischaemic heart disease (IHD) (*n* = 8005)			
Number of admissions (%)	7755 (96.9%)	250 (3.1%)	
Mean age, years (SD)	69.2 (13.2)	63.6 (13.6)	*P* < 0.01^a**^
No. of females (%)	2586 (33%)	82 (33%)	*P* = 0.89^b^
Mean Socio-Economic Index for Area^c^ (SD)	931 (69)	923 (71)	*P* = 0.06^a^
No. of admissions with at least one CC (%)	5475 (71%)	207 (83%)	*P* < 0.01^b**^
Stroke (*n* = 5043)			
Number of admissions (%)	4757 (94.3%)	286 (5.7%)	
Mean age, years (SD)	72.4 (14.7)	64.4 (14.7)	*P* < 0.01^a**^
No. of females (%)	2217 (46%)	119 (42%)	*P* = 0.11^b^
Mean Socio-Economic Index for Area^c^ (SD)	942 (70)	924 (75)	*P* < 0.01^a**^
No. of admissions with at least one additional CC^g^ (%)	2720 (57%)	212 (74%)	*P* < 0.01^b**^

*SD* Standard deviation, *CC* Charlson comorbidity

*The variable differs significantly between the two scenarios at *P* < 0.05.

**The variable differs significantly between the two scenarios at *P* < 0.01.

^a^*P*-value calculated using a t-test

^b^*P*-value calculated using a Fisher exact test

^c^Higher score reflects higher level of advantage and vice versa

^d^Other than the Charlson comorbidity “any malignancy, including lymphoma and leukaemia, except malignant neoplasm of the skin” associated with the primary diagnosis

^e^Other than the Charlson comorbidity “chronic pulmonary disease” associated with the primary diagnosis

^f^Other than the Charlson comorbidity “diabetes without chronic complication” associated with the primary diagnosis

^g^Other than the Charlson comorbidity “cerebrovascular disease” associated with the primary diagnosis

### Comparison of patient characteristics

Patients with mental illness as a comorbidity were significantly younger in comparison to those without mental illness, across the five chronic conditions (Table 2). The average age gap ranged between three to eight years, with the highest age gap in the stroke cohort. There were no significant gender differences between mental illness and without mental illness groups across the cohorts of chronic conditions. Socioeconomic status between the mental illness and non-mental illness groups only differed in the stroke cohort, where patients with mental illness generally belonged to areas of lower socioeconomic advantage.

In the stroke cohort, there was a significantly higher rate of presence of Charlson comorbidity for those with mental illness - 74%, in comparison to 57% for those without mental illness. The presence of a Charlson comorbidity for the patients with cancer and IHD had a similar pattern - 83% and 83% of mental illness, in comparison to 72% and 71% for those without mental illness, respectively.

### Comorbidity of mental illness and LOS variation

Patients with a comorbidity of mental illness had different types of mental illnesses. Table 3 shows blocks of subtype of mental illness ICD-10 codes that occurred in 10 or more admissions, for each chronic condition. Mental and behavioural disorders due to psychoactive substance use was a dominant subtype of mental illness across the five chronic conditions. The other common, but less dominant, subtype of mental illness across the five chronic conditions was neurotic and stress-related disorders.

**Table 3:** Subtypes of mental illness coded in 10 or more admissions of patients, for the five chronic medical conditions

	Cancer	COPD	Diabetes	IHD	Stroke
Total number of patients’ admissions with a diagnosis of mental illness recorded	100	320	90	250	286
F10–F19: Mental & behavioural disorders due to psychoactive substance use	48 (48%)	219 (69%)	47 (52%)	160 (64%)	151 (53%)
F20–F29: Schizophrenia, schizotypal & delusional disorders	< 10	11 (3.4%)	< 10	< 10	< 10
F30–F39: Mood [affective] disorders	< 10	17 (5.3%)	16 (18%)	17 (6.8%)	61 (21%)
F40–F48: Neurotic, stress-related & somatoform disorders	39 (39%)	102 (32%)	16 (18%)	66 (27%)	58 (20%)

Rarer subtypes of mental illness are not listed in the table (e.g. F60 – F69: Disorders of adult personality and behaviour). Percentages in a column can sum to more than 100% because some admissions were coded with more than one type of mental illness

Table 4 reports the association between comorbidity of mental illness and the LOS variation. Within each chronic medical condition, patients with a comorbidity of mental illness had significantly longer LOS than for patients without mental illness (Column 2, Table 4). The column 5 in Table 4 reports the adjusted LOS variation - that is, the percentage increase in mean LOS distinguishably due to a comorbidity of mental illness after controlling for probable confounding variables. In the cohort of cancer and stroke conditions, comorbidity of mental illness unfavourably affected the LOS variation of some patients by as high as 97% and 109%. Though mental and behavioural disorders (due to psychoactive substances) was a dominant subtype of mental illness across the five chronic diseases (Table 3), it contributed a significant and disproportionate unfavourable LOS variation only in the stroke patient cohort i.e. 36.3%.

**Table 4:** LOS variation attributable to mental illness, for each of the five chronic medical diseases

Chronic Disease (number of patients with MI)	Mean LOS in days in scenarios of 1. Without MI (SD) and 2. With MI (SD)	Type of MI^a^ (number of patients with the type of MI)	Mean LOS (SD) in days for patients with this type of MI	Adjusted LOS variation^b^: % increase in mean LOS with MI (95% CI)	Difference in Bed-days’ use^c^	
					Per patient	in 5 years
Cancer (*n*= 100)	Scenario 1: 8.6 (9.3) Scenario 2**: 12.8 (11.8)	F10–F19 Mental and behavioural disorders due to psychoactive substance use (*n* = 48)	9.5 (7.4)	14.6% (–14.3%–53.2%)	NA	NA
		All other subtypes (*n* = 52)	15.8 (14.1)	97.0% (49.9%–159%)	7.8	404
COPD( *n*= 320)	Scenario 1: 4.3 (4.6) Scenario 2*: 4.9 (4.6)	F40–F48 Neurotic, stress-related and somatoform disorders (*n* = 102)	6.2 (5.2)	64.2% (39.4%–93.4%)	2.4	248
		All other subtypes (*n* = 218)	4.3 (4.1)	8.2% (–4.7%–22.9%)	NA	NA
Diabetes (*n* = 90)	Scenario 1: 5.6 (9.3) Scenario 2**: 8.7 (8.5)	All (*n* = 90)	8.7 (8.5)	68.0% (29.6%–118%)	3.5	317
IHD (*n* = 250)	Scenario 1: 3.2 (4.1) Scenario 2**: 6.2 (9.6)	All (*n* = 250)	6.2 (9.6)	90.5% (71.5%–112%)	2.9	735
Stroke (*n*= 286)	Scenario 1: 7.9 (10.8) Scenario 2**: 16.7 (26.7)	F10–F19 Mental and behavioural disorders due to psychoactive substance use (*n* = 151)	12.5 (28.1)	36.3% (16.2%–59.9%)	3.3	505
		All other subtypes (*n* = 135)	21.3 (24.4)	109% (78%–146%)	11.1	1502

*MI* Mental illness, *SD* Standard deviation, *CI* Confidence Interval, *NA* Not applicable

*The variable differs significantly between scenario 1 and 2 at *P* < 0.05

**The variable differs significantly between scenario 1 and 2 at *P* < 0.01

^a^Patients with MI were divided into subtypes if more than 10 admissions were coded with the subtype (see Table 3), and if there was a significant difference in length of stay (LOS) between patients with this subtype of MI and patients with other subtypes of MI. Otherwise, results are presented for all patients with MI grouped together as the case for diabetes and IHD

^b^Percentage increases in LOS, in relation to patients without MI, were estimated after adjusting for the variables: hospital, financial year of patient separation, presence of a Charlson Comorbidity, patient age, patient gender, and socio-economic status (SES) by geographical region

^c^These hypothetical difference in bed-days’ use is calculated with the assumption of same average LOS for patients of chronic medical conditions, between the scenarios of with and without a comorbidity of MI. Difference in bed-days use were calculated based on both MI subtype (if any) and total MI (if no subtype). NA refers to scenarios where hypothetical bed-days’ use was not calculated since no significant increase in adjusted LOS existed

In the cohort of COPD patients, the neurotic, stress-related and somatoform disorders subtype of mental illness markedly increased the unfavourable LOS variation i.e. 64.2%. The diabetes and IHD cohorts did not have any specific sub-type of mental illness that disproportionately increased the LOS.

Comorbidity of mental illness reflected differences in bed days’ use across the chronic medical conditions, when we assumed the adjusted LOS variation between the groups of with and without mental illness to be zero. The greatest difference came from the stroke cohort, with 11.1 days of higher bed use per patient for the mental illness group (“all other subtypes”) than for those without mental illness. The mental illness groups having lower end of differences in bed days’ use than those without mental illnesses were COPD: 2.4 days per patient and IHD: 2.9 days per patient. When we accumulate the scenario for all four hospitals across the chronic medical conditions, the differences in bed days’ use between patients with and without concomitant mental illness over the 5 years was 3,711 (the summation of all the differences in bed days’ use in 5 years, as reported in the last column of Table 4).

## Discussion

This study compared the characteristics of five types of chronic medical condition patients, with and without a comorbidity of mental illness. Furthermore, the study analysed the association between comorbidity of mental illness and the LOS variation of hospitalised patients while controlling for confounding variables. This analysis included assessment of difference in bed days’ use by mental illness patients against those without mental illness, within each chronic medical condition. To the best of the authors’ knowledge, this is unique work investigating the adverse impact of comorbidity of mental illness across a wide range of chronic medical conditions in a state wide public hospital system. Existing literature [41, 42] that reported an association between comorbidity of mental illness and LOS did not concentrate on such a range of chronic conditions. This study addressed the need for greater clarification of the role of mental illness in complex care management [12].

There are two major findings: one, the similarities and distinctiveness of characteristics of patients with and without a comorbidity of mental illness varied across the chronic medical conditions, such as cancer versus diabetes; and, two, the consistent influence of mental illness on unfavourable LOS variation of patients with chronic medical conditions. The degree to which the comorbidity of mental illness influenced the LOS varied across the chronic medical conditions. In certain conditions, such as cancer, COPD and stroke, the influence of comorbidity of mental illness on LOS also varied with subtype of mental illness. These findings support the previously reported association between comorbidity of mental illness and resource utilisation, which could originate from patients’ compromised cognition and involvement in self-care and rehabilitation, or associated complexities such as stigma [12, 41, 42]. This study was not designed to explore the factors that associated length of hospital stay to a comorbidity of mental illness, and future studies will be beneficial in this regard.

This study identified patients with chronic medical condition and a comorbidity of mental illness as being younger than their corresponding non-mental illness cohort. This finding resonated with certain literature [43], but contrasted with others [3, 44]. The 2017 study by Lavin and colleagues [43], which was in the context of acute care end-of-life patients, had similarity with ours in not restricting the study population by age. This is unlike the Adams et al.’s [44] study that restricted the population of the study to 65 years and above. This may infer that studies of patients with a comorbidity of mental illness and chronic medical conditions can identify a relatively younger cohort of patients when there is no age restriction on the study population.

The unfavourable LOS variation for patients with chronic medical conditions, in association with a comorbidity of mental illness, is similar to previous literature [1–5, 19, 44]. For example, Krein et al [2] reported an increase in LOS of around four days in patients with diabetes and a comorbidity of mental illness, which is comparable with our finding of three and a half days difference in bed use. This small difference is perhaps explained by our analysis including a broad scope of mental illnesses (i.e. all of the applicable ICD-10 F codes reported in Table 1), whereas Krein’s research included patients with comorbidities of severe mental illness only. Notwithstanding, the projected differences in bed days use in this study is consistent with the recent literature that reported substantial bed days saving through benchmarking to the average LOS of peer hospitals [7].

### Implications

The findings of this study imply opportunities for common care processes in areas where chronic disease patients with and without mental illness are similar. Application of such common processes will require flexibility in acute care models for patients with chronic medical conditions and a comorbidity of mental illness. This need for flexibility aligns with current policy guidance for integrated and patient-centred care with mental health and multimorbidity conditions [8, 11, 45, 46]. Joint health and behavioural risk education for common issues between the two cohorts seems an efficient strategy [10, 11]. In areas where the two cohorts are different, such as the significant age differences found across the five chronic diseases in this study, distinctive care processes that accommodate age-specific risk factors are applicable [47]. These distinctive processes should be about integrating care for medical and mental illnesses for a relatively younger cohort, so that care for this cohort does not become more complex with progressing age [48]. Therefore, health professionals will strengthen the tasks of integration and patient centeredness if they are capable of switching between common and distinctive care processes. We understand the switching between common and distinctive processes will also require upskilling of healthcare teams. This upskilling will potentially include unlearning the rigidity of strict clinical specialism [49]. Additionally, health professionals will need to rely on collaboration skills for practices such as effective engagement between general hospital staff and psychiatric liaison teams [50].

The reported LOS variation highlights the necessity for LOS improvement strategies for chronic disease patients with a comorbidity of mental illness. Hospitals will save substantial bed-days by levelling the bed days’ use of this vulnerable population to that of patients only with chronic medical conditions. However, the LOS improvement strategies should work towards improved care in a realistic manner. A portion of the differences in bed days’ use will not usually translate to reduced cost or be available for use, accommodating for contingent bed capacity and required quality of care [51, 52]. LOS efficiency practices need to consider a hospital’s mix of different types of chronic diseases and comorbidities of subtype of mental illness. For example, in our study, COPD and diabetes showed similar differences in bed days’ use over five years, despite diabetes having one bed day higher difference per patient - as Tasmanian hospitals manage higher number of admissions of patients with a comorbidity of mental illness and COPD. The study found that the same subtype of mental illness (mental and behavioural disorders due to psychoactive substance use) influenced bed days’ use in a varied manner between chronic medical conditions such as cancer and stroke. Hence, a hospital’s mix of comorbidities of medical and mental illnesses will influence efficiency gains from different LOS efficient practices, such as better planning of discharges and patient flow [53]. Findings in our study confirm that a relatively small health system, catering to a population of about 500,000, could make substantial savings in bed days, from LOS efficient practices with chronic medical disease patients with comorbidity of mental illness. Again, a challenge is upskilling of healthcare teams [54], for quality sensitive implementation of initiatives directed at bed days saving.

Another implication is the need for policy guidance that facilitates routine reporting and monitoring of LOS variation between chronic medical condition patients with and without mental illness. Open evidence of unfavourable LOS variation between peer hospitals should empower health professionals to develop strategies to minimise any unwarranted variation [55, 56]. Government-led initiatives, such as the OECD health care variation study and the Australian atlas of healthcare variation, are examples in this regard [15, 57, 58]. Increased transparency about the influence of mental illness on LOS variation can strengthen evidence for chronic disease and mental health service research, contributing to better system-based approaches for multimorbidity and patient equity in the long-term [59].

There were a few study limitations, particularly regarding availability of data. For example, the SES of admitted patients was a generalisation based on the average socio-economic status of the area of residence of individuals, and individuals can have different socio-economic status to that of the area average. We may not have captured all acute patients of comorbidities of physical and mental illness, as there can be misses in coding of a diagnosis of mental illness in patients admitted for chronic medical conditions [60]. The adjusted LOS variation data in this study accounted for six probable confounding variables but there could be more confounding factors, such as a patient’s use of mental illness services prior to hospital admission.

## Conclusion

Patients with and without a comorbidity of mental illness can have similar and distinctive characteristics across primary medical conditions of cancer, COPD, diabetes, IHD and stroke. Comorbidity of mental illness consistently produced unfavourable LOS variation across these five medical conditions. There is need for improved integrated care models and efficiency in LOS for patients with chronic medical conditions and comorbidity of mental illness. Given that this vulnerable population is growing, we need greater policy guidance for widespread reporting and analysis of LOS variation of this patient cohort. Upskilling of associated healthcare teams is also a necessity in this regard. We hope this study will generate interest for future studies about ways to attain the same LOS for acute care patients with chronic medical conditions, regardless of their mental health status.

## Abbreviations

CC: Charlson comorbidity
CI: Confidence interval
COPD: Chronic obstructive pulmonary disease
ICD: International classification of disease
IHD: Ischaemic heart disease
IRSAD: Index of relative socio-economic advantage and disadvantage
LOS: Length of stay
MI: Mental illness
